# Tea Plants With Gray Blight Have Altered Root Exudates That Recruit a Beneficial Rhizosphere Microbiome to Prime Immunity Against Aboveground Pathogen Infection

**DOI:** 10.3389/fmicb.2021.774438

**Published:** 2021-12-01

**Authors:** Qiaomei Wang, Ruijuan Yang, Wenshu Peng, Yanmei Yang, Xiaoling Ma, Wenjie Zhang, Aibing Ji, Li Liu, Pei Liu, Liang Yan, Xianqi Hu

**Affiliations:** ^1^College of Plant Protection, Yunnan Agricultural University, Kunming, China; ^2^Puer Institute of Pu-Erh Tea, Pu’er City, China; ^3^College of Pu’er Tea, West Yunnan University of Applied Sciences, Pu’er City, China; ^4^College of Food Science and Technology, Yunnan Agricultural University, Kunming, China; ^5^School of Biological and Chemical Science, Pu’er University, Pu’er City, China

**Keywords:** tea gray blight, foliar pathogen, rhizosphere microbiome, root exudates, induced systemic resistance

## Abstract

Tea gray blight disease and its existing control measures have had a negative impact on the sustainable development of tea gardens. However, our knowledge of safe and effective biological control measures is limited. It is critical to explore beneficial microbial communities in the tea rhizosphere for the control of tea gray blight. In this study, we prepared conditioned soil by inoculating *Pseudopestalotiopsis camelliae-sinensis* on tea seedling leaves. Thereafter, we examined the growth performance and disease resistance of fresh tea seedlings grown in conditioned and control soils. Next, the rhizosphere microbial community and root exudates of tea seedlings infected by the pathogen were analyzed. In addition, we also evaluated the effects of the rhizosphere microbial community and root exudates induced by pathogens on the performance of tea seedlings. The results showed that tea seedlings grown in conditioned soil had lower disease index values and higher growth vigor. Soil microbiome analysis revealed that the fungal and bacterial communities of the rhizosphere were altered upon infection with *Ps. camelliae-sinensis.* Genus-level analysis showed that the abundance of the fungi *Trichoderma*, *Penicillium*, and *Gliocladiopsis* and the bacteria *Pseudomonas*, *Streptomyces*, *Bacillus*, and *Burkholderia* were significantly (*p* < 0.05) increased in the conditioned soil. Through isolation, culture, and inoculation tests, we found that most isolates from the induced microbial genera could inhibit the infection of tea gray blight pathogen and promote tea seedling growth. The results of root exudate analysis showed that infected tea seedlings exhibited significantly higher exudate levels of phenolic acids and flavonoids and lower exudate levels of amino acids and organic acids. Exogenously applied phenolic acids and flavonoids suppressed gray blight disease by regulating the rhizosphere microbial community. In summary, our findings suggest that tea plants with gray blight can recruit beneficial rhizosphere microorganisms by altering their root exudates, thereby improving the disease resistance of tea plants growing in the same soil.

## Introduction

The soil near plant roots, i.e., the rhizosphere, plays a key role in plant immunity and overall plant performance (Pineda et al., 2020) and in protecting above-ground plant tissues from pests and diseases (Edwards et al., 2019). Some rhizosphere soils can inhibit the occurrence of plant diseases even in the presence of virulent pathogens or under climatic conditions conducive to disease development (Weller et al., 2002; Mazzola, 2004). In such disease-suppressive soils, disease inhibition is related to the abundance of beneficial microorganisms (Mendes et al., 2011; Cha et al., 2016). While some beneficial microorganisms directly inhibit pathogens by producing antibacterial compounds, others indirectly inhibit pathogens by stimulating the plant immune system, a phenomenon termed induced systemic resistance (ISR) (Pieterse et al., 2014). *Pseudomonas simiae* WCS417 is probably the best-characterized microbe capable of eliciting ISR (Lundberg and Teixeira, 2018). It has been suggested that disease-suppressive soils are a consequence of the accumulation of beneficial microorganisms caused by repeated pathogen infection of aboveground plant parts (Schlatter et al., 2017; Yuan et al., 2018). *Arabidopsis thaliana* plants infected with the downy mildew pathogen recruited beneficial microorganisms that induced disease resistance and promoted growth, thus improving the survival chances of offspring growing in the same soil (Berendsen et al., 2018). Similarly, aphid and whitefly infestations led to the recruitment of beneficial bacteria that helped pepper plants cope with subsequent pathogen attacks (Lee et al., 2012; Kong et al., 2016).

In this process, root exudates are considered to play a regulatory role. During plant growth, the root system continuously releases root exudates, which are complex mixtures of soluble organic substances, into the rhizosphere (Zhao et al., 2015). Root exudates attract or inhibit specific microbial species to accumulate in the rhizosphere, forming a specific rhizosphere microbial community (Gu et al., 2016; Lundberg and Teixeira, 2018). Rhizosphere species and contents change with plant species and are closely related to plant health (Badri et al., 2013). For example, infection of *A. thaliana* leaves by *Pseudomonas syringae* pv. *tomato* increased the secretion of malic acid from the roots and enriched *Bacillus subtilis* in the rhizosphere (Rudrappa et al., 2008). In tomato, the presence of foliar pathogens increased the secretion of caffeic acid, leading to the reshaping of the rhizosphere microorganisms (Gu et al., 2016). *P. syringae* pv. *tomato-*infected *A. thaliana* recruited beneficial rhizosphere communities by secreting long-chain organic acids (Yuan et al., 2018). These findings suggest that plants can alter their root exudates when attacked to recruit beneficial microorganisms to generate a “soil memory” that benefits future plant performance in the same soil. However, the interaction mechanisms between different plants and pathogens are completely different. Studying the interaction mechanisms in different crops and their main pathogens will provide support for sustainable agricultural development (Kaplan et al., 2018; Mariotte et al., 2018).

Tea [*Camellia sinensis* (L.) O. Kuntze] is an important cash crop that plays an important economic role in planting areas (Arafat et al., 2017). Thus, improving tea yield and quality has an important economic impact (Wang et al., 2016). Tea gray blight is one of the most serious foliar diseases of tea plants (Wang et al., 2019). It is caused by *Pseudopestalotiopsis* spp., which severely affect tea yield and quality (Chen et al., 2017b). The disease mainly affects mature and old foliage, but can also affect young shoots (Maharachchikumbura et al., 2014). The harm of the disease is generally aggravated under high temperature and humidity, resulting in poor plant growth and decreased tea yields (Wang and Lu, 2008). At present, chemical pesticides are mainly used in the prevention and control of tea gray blight. However, these are associated with serious problems, including drug resistance in pathogens and pesticide residues (Hong et al., 2005). Although some progress has been made in the screening of antagonistic strains of tea gray blight (Hong et al., 2005, 2006; Li et al., 2010), little is known about sustainable agricultural control measures.

In this study, we aimed to investigate whether tea plants with aboveground *Pseudopestalotiopsis camelliae-sinensis* infection can recruit beneficial rhizosphere microbes by altering their root exudates to resist subsequent pathogen infection. Tea leaves were infected with *Ps. camelliae-sinensis*, and the microbial communities in the rhizosphere and bulk soils were evaluated by high-throughput 16S rRNA and ITS rRNA gene tag sequencing to evaluate whether infection of the aboveground plant parts would change the composition of rhizosphere microorganisms. Root exudates from infected and non-infected tea seedlings were collected and analyzed by LC-MS/MS to evaluate whether the exudate profile was changed after challenge with *Ps. camelliae-sinensis*. We next tested whether these compounds would affect plant resistance by regulating microorganisms. Finally, culturable beneficial microorganisms were isolated from the conditioned soil, and their abilities to induce disease resistance and promote plant growth were measured. Through these combined lines of investigation, we were able to explore how tea gray blight affects the tea plant root exudates to recruit beneficial microorganisms to the rhizosphere and induces systemic resistance to above-ground pathogens in tea plants.

## Materials and Methods

### Isolation and Identification of the Pathogen Causing Tea Gray Blight

Infected tea leaves were collected from tea plantations in Pu’er City, Yunnan, China. Pathogenic fungi were isolated from the infected tea leaves according to previous methods (Chen et al., 2017b), with some modifications. Briefly, tea leaves with symptom spots of tea gray blight were cut into two 4-mm pieces, surface-sterilized with 75% alcohol for 1 min, washed with sterile water, disinfected with 1% sodium hypochlorite for 3 min, and finally washed with sterile water three times. Small pieces were placed on potato dextrose agar (PDA) plates containing 100 μg ml^–1^ chloramphenicol. After 3 days of dark culture at 28°C, single mycelia were selected and transferred to PDA plates for dark culture at 28°C for 7–10 days. The isolated and purified strains were identified using morphological and molecular biological methods (Keith et al., 2006). Briefly, the size (length and width) of conidia, intermediate cells, and apical and basal appendages were measured, and the color of the conidia and the number of apical and basal appendages were observed under a light microscope. For each strain, 30 conidia were observed. The strains were further identified with molecular methods. Briefly, genomic DNA was extracted from fresh mycelium of cultures grown on PDA for 7 days using the CTAB method according to the procedures reported by Than et al. (2008) and Chen et al. (2017a). Three pairs of primers (ITS, β-tubulin, and TEF) were used for PCR amplification. The primers are listed in Supplementary Table 1. The amplified products were separated on a 1% agarose gel and purified using the Gel Extraction Kit (Omega Bio-Tek, Norcross, GA, United States) according to the manufacturer’s protocol. The purified PCR products were cloned into the pMD ^®^19-T Vector (Takara, Japan). Positive transformants were sequenced at Shanghai Sangon Biotech (Shanghai, China). Neighbor-joining trees were constructed using MEGA 5.0 (Kumar et al., 2016).

The pathogenicity of the strains was determined on tea plants. Pathogen cakes with a diameter of 6 mm were placed on tea leaves with or without premade wounds with the mycelial side facing down and covered with sterile absorbent cotton to maintain a moist environment. Tea leaves with wounds covered with sterile PDA plugs were used as controls. Tea plants were randomly assigned to the different treatments, and after treatment, they were transferred to and randomly placed in the greenhouse. The humidity in the greenhouse was maintained above 80% to facilitate pathogen infection. Seven days after inoculation, pathogens were isolated from all tea leaves with symptomatic lesions.

### Development of Conditioned Soils

The soil used in the study was collected in Pu’er City (22.48°N, 100.58°E; altitude, 1,390 m) in September 2019 at a tea plantation where Yunkang No. 10 tea plants have grown for 35 years. The surface soil (10–15 cm) was removed, and the layer between 15 cm and 30 cm was collected as natural soil (NS). The soil was transported to the laboratory, dried at room temperature, and sieved to remove debris (Yuan et al., 2018). The NS had the following characteristics: pH 4.8, available phosphate 8.6 mg kg^–1^, available potassium 65 mg kg^–1^, alkali-hydrolysable nitrogen 38.4 mg kg^–1^, and organic matter 37,000 mg kg^–1^ soil. The soil was sterilized at 121°C for 2 h and transferred to sterile bags (6.0 cm × 6.0 cm × 12.0 cm) that were placed on seedling nursing trays (24 bags per tray).

Woody branches were cut from the tea plants, and each node was cut into a cutting (∼8 cm long), with one leaf preserved per cutting. The cuttings were immersed in 0.1% carbendazim and 0.1% rooting powder for 4 h and then planted in the bags on the seedling nursing trays (1 cutting per bag). All trays were sealed and arranged in a single greenhouse (28°C ± 2°C, 12-h/12-h light/dark cycle) and watered every 2 weeks.

After 18 months of growth, the seedlings were transferred to 36 pots (13 cm × 13 cm × 20 cm, 18 replicates per treatment) containing 3 kg of soil each. Plants in half of the pots (18 pots) were inoculated with *Ps. camelliae-sinensis* at 2 weeks post-transplantation. Four true leaves of each plant were cut with a sterilized blade, and a fungal cake with a diameter of 6 mm was put on the wound, which was then covered with sterile absorbent cotton to maintain a moist environment. The soil was covered with a plastic film to avoid contamination by *Ps. camelliae-sinensis*. Leaves of plants in the remaining 18 pots were wounded by cutting, and the wounds were covered with a sterile PDA plug as controls. Plants were randomly assigned to the treatments, and after treatment, the pots were transferred to and randomly placed in the greenhouse. The humidity in the greenhouse was maintained above 80% to facilitate pathogen infection.

*Ps. camelliae-sinensis* infection was allowed to develop for 10 days until the obvious round disease spot appeared on the tea leaves. Then, the aboveground parts of the tea plants were removed. Part of the rhizosphere and bulk soils were used for microbial diversity analysis, and the remaining soil was used to evaluate the feedback relationship between aboveground pathogen infection and the soil.

### Soil Sample Collection and Soil Chemical Analyses

Rhizosphere soil was collected according to a previously reported method (Luo et al., 2019), with some modifications. The roots of the tea plants were dug out and the loose soil on the root surfaces was removed. The remaining soil within 1 mm from the root surface was collected as rhizosphere soil. Briefly, the roots were placed in a sterile tube containing 40 mL of 1× phosphate-buffered saline (PBS). After centrifugation at 12,000 rpm for 15 min, the precipitate was collected as rhizosphere soil. The soil left in the pot after the removal of the plants was defined as bulk soil. Six biological replicates of each treatment (24 samples) were collected and stored at −80°C until use. After removing the aboveground plant parts, 5 g bulk soil was collected from the conditioned and control soils for soil chemical analysis. The contents of available phosphorus (AP), available potassium (AK), nitrate (NO_3_^–^), and ammonia (NH_4_^+^), and pH were determined according to Bakker et al. (2015).

### Sequence Analysis of the Complete ITS and 16S rRNA Genes

Total DNA was extracted from the soil samples using the PowerSoil ^®^ DNA isolation Kit (MO BIO Laboratories, Carlsbad, CA, United States) according to the manufacturer’s instructions. DNA quality was evaluated using a NanoDrop 1000 spectrophotometer (Thermo Fisher Scientific, Wilmington, MA, United States), and the purified DNA was stored at −80°C until use. The complete fungal ITS and bacterial 16S rRNA genes were amplified from the total soil DNA using the ITS1/ITS4 and 27F/1492R primers, respectively, and sequenced using the PacBio platform at BIOMARKER (Beijing, China). PacBio Sequel subreads were demultiplexed and assembled into circular consensus sequences using SMRT link version8.0). The LIMA (v1.7.0) software was used to identify barcode sequences and remove chimeras to obtain high-quality consensus sequences (Callahan et al., 2016). QIIME2 (v2020.6) was used to denoise the data after quality control, using an amplicon sequence variant threshold of 0.005%. After normalization, minimum data for all samples were subjected to community richness and diversity analyses (based on the alpha diversity index) and principal coordinate analysis (PCoA) in QIIME2.

### Evaluation of the Effect of Conditioned Soil on Plant Performance

After the conditioned and control soils were dried for 1 week, 200 mL of Hoagland solution was added, and 18-month-old tea seedlings were transplanted into the soils. The pots were placed randomly in the greenhouse for cultivation. After 15 days, half of the seedlings were inoculated with *Ps. camelliae-sinensis* as described above. Seven days after inoculation, the size of spots was measured to calculate the disease incidence grade and disease index (DI). The incidence grade was calculated according to the formula in Supplementary Table 2 (Wang and Lu, 2008; Wu et al., 2015), and the DI was calculated as follows:


$$\text{DI}=\frac{\sum \left(\mathrm{number}\mathrm{of}\mathrm{diseased}\mathrm{plants}\mathrm{in}\mathrm{this}\mathrm{index}\times \mathrm{di}\right)}{\left(\mathrm{total}\mathrm{number}\mathrm{of}\mathrm{plants}\mathrm{investigated}\times \mathrm{highest}\mathrm{di}\right)}\times 100\%.$$

For the half of the tea seedlings that were not inoculated with the pathogen, plant height was measured at 15 and 45 days after transplantation. The growth rate of the seedlings over 30 days was calculated to evaluate the effect of conditioned soil on growth.

### Root Exudate Collection and Ultra-High Performance Liquid Chromatography-Tandem Mass Spectrometry Analysis

Tea root exudates were collected as previously described (Luo et al., 2020), with some modifications. Soil attached to the roots of healthy tea seedlings was gently rinsed off with sterile water, and the seedlings were transplanted into hydroponic pots containing 3 kg sterilized quartz sand that had been soaked in 10% HCl for 2–3 h, eluted with deionized water until the pH was stable at 6.5–7.0, and then sterilized. The pots were randomly placed in the greenhouse at a temperature of 28°C in the day and 23°C at night, and a 12-h/12-h light/dark cycle.

Tea seedlings were soaked in sterile water for 2 weeks and irrigated with 100 mL of Hoagland solutions every 3 days. Then, they were inoculated with *Ps. camelliae-sinensis* as described above and were transferred to glass bottles containing 500 mL of sterile water. The bottles were randomly placed in the greenhouse during the exudate collection period. Each treatment comprised three replicates, including six tea seedlings, and leaves inoculated with sterile PDA medium were used as controls. After incubation for 5 days, the liquid in six glass bottles was combined into a single sample and was defined as the root exudate. The collected liquid samples were filtered through a filter paper and 0.22-μm hydrophilic membrane.

The root exudates were extracted from the aqueous solution with 70% methanol and analyzed by UPLC-MS/MS as reported previously (Hu et al., 2018). Briefly, UPLC normal-phase chromatography was performed using a SHIMADZU Nexera X2, and MS data were collected using a 4500 QTRAP MS. Compounds were separated on an Agilent SB-C18 column (2.1 × 100 mm i.d., 1.8-μm particle sizes). Water (0.1% formic acid) and acetonitrile (0.1% formic acid) were employed as mobile phases A and B. The elution profile was: 0–9 min, 95–5% A in B; 9–10 min, 95% B; 10–10.1 min 5–95% A in B, 10.1–14 min 5% B. The mobile phase flow rate was 0.35 mL/min. The column temperature was maintained at 40°C, and the injection volume was 4 μL. In the AB 4500 QTRAP UPL-MS/MS system (AB SCIEX, CA, United States), the electrospray ionization temperature was 550°C, the capillary voltage was 5,500 V (positive ion mode)/−4,500 V (negative ion mode), and the curtain gas pressure was 50, 60, and 25 psi. All other MS parameters were left at their default values as suggested by the manufacturer. Data were analyzed using Analyst v1.6.3 (AB Sciex).

### Preparation of Exudate-Conditioned Soils Using Exogenous Root Exudates

To investigate the effect of root exudates on soil microbial-mediated soil–plant feedback, we added exogenous root exudates to rhizosphere soil to prepare exudate-conditioned soils to evaluate the feedback effect of soil microorganisms on plant disease. The method used was modified from a previous report (Yuan et al., 2018). Briefly, we focused on four types of secretions: amino acids, flavonoids, phenolic acids, and alkaloids. Representative compounds of each exudate category were selected based on their abundance in root exudates after *Ps. camelliae-sinensis* infection. Amino acids represented the pathogen-repressed root exudates, and flavonoids, phenolic acids, and alkaloids represented the pathogen-induced root exudates. The final concentration of the mixture solutions in each group was 10 mM. Mixture solutions of pathogen-repressed exudates contained 2.0 mM L-glycyl-L-proline, 2.0 mM L-valine, 2.0 mM L-methylpiperidine-2-carboxylic acid, 2.0 mM L-tryptophan, and 2.0 mM 2-aminoisobutyric acid. Mixture solutions of pathogen-induced exudates contained 2.0 mM butin, 2.0 mM epicatechin, 2.0 mM paeonol, 2.0 mM 2′,4′,6′-trihydroxyacetophenone, and 2.0 mM putrescine.

Six hundred grams of soil was placed in pots, which were incubated at 30°C for 1 week to preculture the soil microorganisms. Then, the pots were randomly placed in a greenhouse at 28°C, and 60 mL of mixture solution was poured into the pots twice a week for 8 weeks (16 times in total). Three treatments were designed as follows: (1) pathogen-repressed (PR) group, (2) pathogen-induced (PI) group, and (3) water control (WC) group. Each treatment comprised three replicates.

### Evaluation of the Effect of Exudate Compounds on Aboveground Plant Disease

After preparation and filtration as reported previously (Yuan et al., 2015), soil slurries were applied to 18-month-old tea seedlings to investigate their effect on the soil microbiome. Briefly, to prepare soil slurries, 350 g of exudate-conditioned soil was mixed with 3.5 L of sterile water and shaken on an orbital shaker for 1 h. The mixture solutions were allowed to settle for 1 h, and the supernatant was centrifuged at 9,000 rpm for 10 min. The supernatant was defined as the soil slurry suspension containing microorganisms. Half of the suspensions were centrifuged again at 12,000 rpm for 15 min and then filtered through a 0.22-μm filter to remove most of the soil microorganisms. Filter-sterilized slurries were used as controls. Slurries were prepared from PR soil, PI soil, WC soil, and NS and mixtures of PR and PI soils at ratios of 9:1, 5:5, and 1:9 (v/v). Eighteen-month-old tea seedlings (prepared as described above) were transplanted into pots containing sterilized soil and 300 mL of unfiltered soil slurry, filtered and sterilized soil slurry, or sterile water. Three days after transplantation, 100 mL of Hoagland solution was added to provide nutrients for the growth of the tea seedlings. Two weeks later, *Ps. camelliae-sinensis* was inoculated onto the tea leaves as described above. Disease incidence in the tea seedlings was calculated 7 days after infection.

### Isolation and Identification of Culturable Fungi and Bacteria in Conditioned Soil

Rhizosphere soil was collected as described above, and 10 g soil was added to 90 mL of PBS. The soil suspension was homogenized for 15 min, diluted from 10^–1^ to 10^–5^ (Yang et al., 2020), and 0.1 mL of each diluent was added to nutrient agar medium (NA, containing per L of distilled water: 15 g peptone, 5 g NaCl, 3 g beef extract, 15 g agar; pH 7) and rose Bengal medium (RBM, containing per L of distilled water: 5 g peptone, 10 g glucose, 1 g KH_2_PO_4_, 0.5 g MgSO_4_.7H_2_O, 15 g agar, 100 mL 1/3,000 rose Bengal solution, 0.1 g chloramphenicol) for bacterial and fungal culture, respectively (Zhou et al., 2014; Tchakounté et al., 2018). The NA and RBM plates were placed in incubators at 37 and 28°C, respectively, and incubated for 2–5 days. Then, single colonies were picked and inoculated onto NA (bacteria) or PDA (fungi) to obtain pure cultures, which were stored at 4°C until use. For strain identification, please refer to the method in sub-section “Isolation and Identification of the Pathogen Causing Tea Gray Blight.” The primers used have been listed in Supplementary Table 3.

### Screening of Beneficial Microbes in the Rhizosphere and Their Inhibitory Effect on the Pathogen Causing Tea Gray Blight

Microorganisms belonging to *Trichoderma*, *Gliocladiopsis*, *Penicillium*, *Streptomyces*, *Bacillus*, *Burkholderia*, and *Pseudomonas* with significantly increased abundance in conditioned soil were selected from the isolated and identified culturable strains. The selected beneficial strains were paired cultured with *Ps. Camelliae-sinensis*, and their inhibition percentage against the pathogen growth was tested (Liu et al., 2020). Briefly, a pathogen mycelium cake (6 mm diameter) was placed in the center of a PDA plate, and four culturable microbial cakes were placed around the pathogen at equal distances (20 mm). PDA plates with only the pathogen were used as control. Each treatment was repeated three times. All plates were cultured at 28°C for 3 days. The pathogen colony diameter was measured, and the inhibition rate of the culturable microbes on the growth of pathogen was calculated as follows:


$$\begin{array}{c} \mathrm{Growth}\mathrm{inhibition}\mathrm{rate}\left(\%\right)=100\\ \times \frac{\left(\mathrm{radial}\mathrm{growth}\mathrm{of}\mathrm{control}-\mathrm{radial}\mathrm{growth}\mathrm{of}\mathrm{treated}\mathrm{sample}\right)}{\mathrm{radial}\mathrm{growth}\mathrm{of}\mathrm{control}}. \end{array}$$

### Evaluation of the Beneficial Microbes in Promoting the Growth of Plants and Alleviating Aboveground Disease

A pot experiment was used to evaluate the effects of the beneficial microbes in promoting tea seedling growth and inhibiting aboveground pathogen infection. The soil used was NS collected from the tea garden it was placed in pots (13 × 13 × 20 cm), and two 18-month-old tea seedlings were transferred in each pot. After 15 days, the pots were irrigated with either 300 mL (10^8^ cfu mL^–1^) suspension of the beneficial microbes or the same volume of sterile water as a control. Three days later, half of the tea seedlings were inoculated with *Ps. camelliae-sinensis*. Seven days after infection, the diameter of disease spots was measured to calculate the disease incidence grade and DI (inoculation and culture conditions were the same as described above). The remaining half of the tea seedlings were irrigated with the solution of beneficial microbes solutions and were allowed to grow for 30 days. Thereafter, their height was measured to evaluate the effect of the beneficial microbes on the growth of the tea seedlings. All treatments comprised three replicates, including six seedlings each.

### Statistical Analysis

SPSS v26.0 (IBM SPSS Inc., Chicago, IL, United States) was used for data analysis. Before statistical analysis, data normality and homogeneity of variance were tested. Differences among the treatments were analyzed by one-way analysis of variance and Duncan’s multiple-range test.

## Results

### Pathogen Causing Tea Gray Blight

**FIGURE 1:**
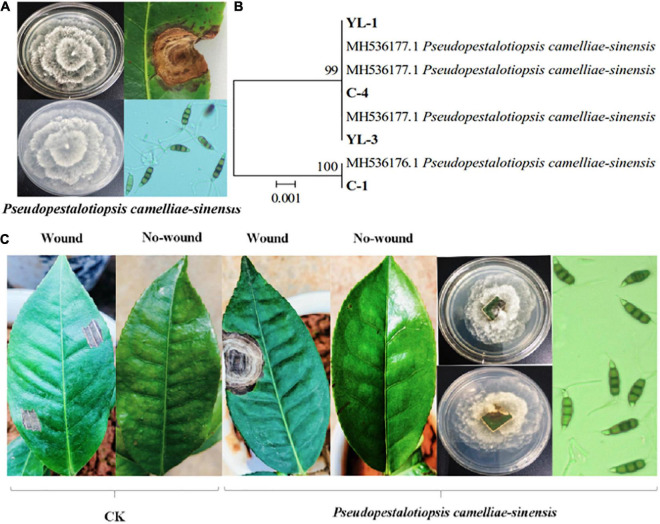
Identification of the pathogen causing tea gray blight and pathogenicity tests. Colony morphology of, symptoms caused by, and conidia of *Ps. camelliae-sinensis.* **(A)** Hierarchical clustering based on β-tubulin gene sequences of pathogens **(B)**. *In-vitro* pathogenicity tests of tea leaves **(C)**.

Ten strains of fungi were isolated from tea leaves showing typical symptoms of tea gray blight (i.e., leaf spots). Four of the strains showed obvious pathogenicity to tea leaves. Based on colony morphology, conidia characteristics, and ITS, β-tubulin, and TEF sequences, the four strains were identified as *Ps. camelliae-sinensis* (Figures 1A,B). When the identified strains were re-inoculated on tea leaves, 7 days later, they produced the typical tea gray blight spots on the leaves. Most of the strains did not produce any symptoms on non-wounded tea leaves. Control tea leaves with sterile PDA medium did not show any symptoms of tea gray blight, and *Ps. camelliae-sinensis* could not be isolated from these leaves (Figure 1C).

### Effect of Conditioned Soil on Plant Performance

**FIGURE 2:**
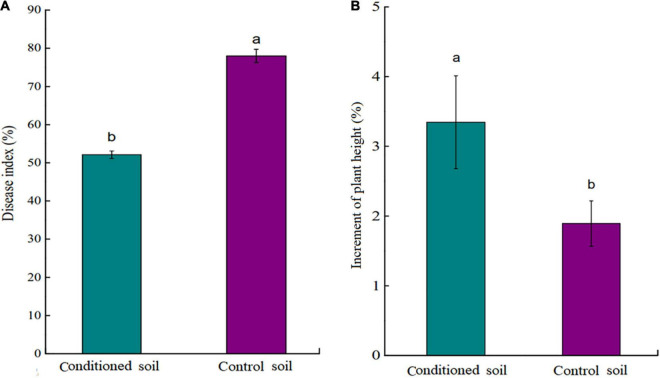
**(A)** Disease index (DI) of tea seedlings grown in conditioned or control soil and challenged with *Ps. camelliae-sinensis* for 7 days. **(B)** Plant height of tea seedlings grown in conditioned or control soil for 30 days. a and b represent statistically significant differences between treatments as determined by Student’s *t*-test (*p* < 0.05). Data are means ± SEs (*n* = 6).

The results indicate that tea seedlings grown in conditioned soils showed a significantly lower DI than those grown in control soil (*p* < 0.05; Figure 2A). In the absence of *Ps. camelliae-sinensis*, tea seedlings grown in conditioned soil showed significantly stronger growth than plants grown in control soil (*p* < 0.05; Figure 2B). This indicated that the conditioned soil could promote the growth of the tea seedlings. In addition, there was no significant (*p* > 0.05) difference in pH and nutrient content (NH_4_^+^, NO_3_^–^, AP, and AK) between conditioned and control soil (Supplementary Table 4). The data showed that nutrient availability did not drive differences in tea seedlings growth in soil regulation.

### Effects of Aboveground Pathogen Infection on Soil Fungal and Bacterial Communities

**FIGURE 3:**
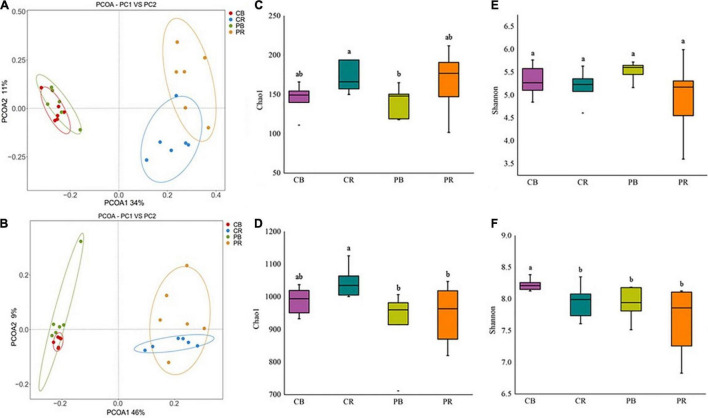
Principal coordinate analysis (PCoA) of fungi **(A)** and bacteria **(B)**, community abundance and diversity indices of fungi **(C,E)** and bacteria **(D,F)** in conditioned and control soils. CB and CR represent control (C) bulk (B) and rhizosphere (R) soil, respectively. PB and PR represent conditioned (P) bulk (B) and rhizosphere (R) soil, respectively. Data are means ± SEs. a and b indicate significant differences among the soils (*p* < 0.05).

Rhizosphere and bulk soils of conditioned and control soils were analyzed by PacBio sequencing. In total, 17,877 fungal OTUs and 18,179 bacterial OTUs were obtained for the analysis of the fungal and bacterial communities, respectively (Supplementary Tables 5, 6). PCoA results based on the Bray-Curtis statistic showed that the community structures of fungi and bacteria in rhizosphere soil were significantly different from those in bulk soil (Figures 3A,B). The difference in the microbial communities between conditioned and control bulk soils was small, whereas that between conditioned and control rhizosphere soils was larger (Figures 3A,B). Fungal abundance and diversity did not show significant differences between conditioned and control soils (Figures 3C,E). Compared to that in control soil, the bacterial abundance was significantly decreased in conditioned soil (Figure 3D), whereas the bacterial diversity did not show significant differences (Figure 3F and Supplementary Figures 1A–D).

### Microbial Composition of Conditioned Soil

Aboveground pathogen infection altered the fungal and bacterial communities in the rhizosphere soil of tea seedlings at the phylum level (Supplementary Figures 2–4). As for rhizosphere fungi, the relative abundance of Ascomycota was significantly increased (*p* < 0.01), whereas that of Mucoromycota was significantly suppressed (*p* < 0.01) by aboveground pathogen infection (Supplementary Figure 2). In the bulk soil, the relative abundances of Ascomycota, Basidiomycota, and Glomeromycota were significantly increased (*p <* 0.05), whereas that of Chytridiomycota was significantly decreased (*p* < 0.05) (Supplementary Figure 3). For rhizosphere bacteria, seven phyla were significantly affected by aboveground plant infection; the relative abundances of Proteobacteria and Gemmatimonadetes were significantly increased (*p* < 0.05), whereas those of Patescibacteria, Armatimonadetes, Planctomycetes, Bacteroidota, and Verrucomicrobia were significantly decreased (*p* < 0.05) (Supplementary Figure 4). In the bulk soil, there were no significant differences in bacteria at the phylum level.

**FIGURE 4:**
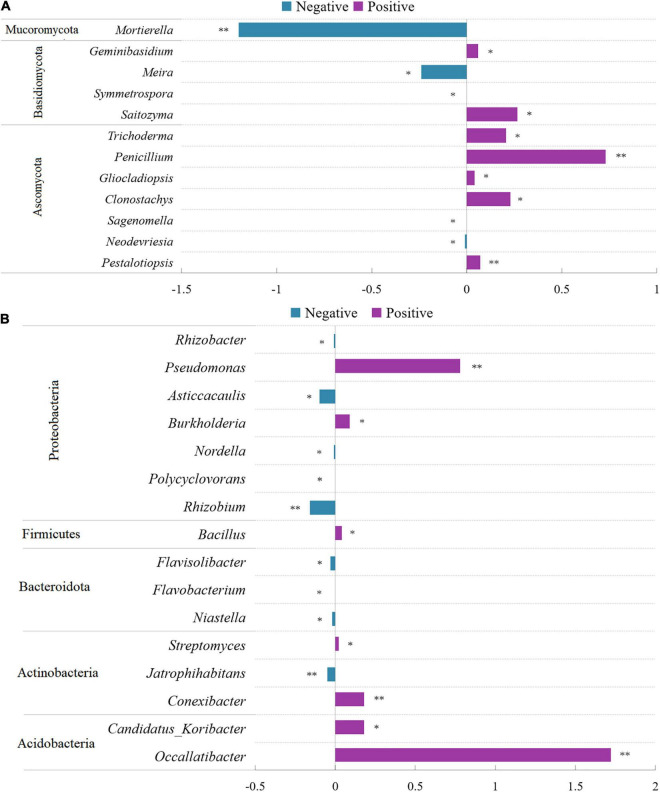
Comparison of fungi **(A)** and bacterial **(B)** abundance differences between conditioned and control rhizosphere soils at the genus level. Data are means ± SEs. *p < 0.05, ^**^p < 0.01 vs. control soil.

At the genus level, 12 fungal genera showed significant differences between control and conditioned rhizosphere soils (Figure 4A). Compared with those in the control rhizosphere soil, the relative abundances of two genera of Basidiomycota were significantly increased, and two genera were significantly decreased in response to above-ground pathogen infection (Figure 4A). Five genera of Ascomycota were induced by aboveground pathogen infection, whereas two genera were inhibited (Figure 4A). Among them, *Trichoderma*, *Penicillium*, and *Gliocladiopsis*, which have biocontrol potential, were enriched in the conditioned rhizosphere soil, whereas, *Mortierella*, an important fungus promoting the transformation of soil nutrients, was inhibited by aboveground pathogen infection. As for bulk soil fungi, three phyla, including 15 genera, showed significant alterations; the relative abundances of nine genera were significantly decreased in conditioned soil, whereas six genera were significantly increased (Supplementary Figure 5). The effects of aboveground pathogen infection on beneficial and harmful fungi in bulk soil were not significant.

As for rhizosphere bacteria, 16 bacterial genera showed significant differences between control and conditioned soils. They belonged to Acidobacteria, Actinobacteria, Bacteroidota, Firmicutes, and Proteobacteria. The relative abundances of seven genera were significantly increased, whereas nine genera were decreased in response to aboveground pathogen infection (Figure 4B). Of these genera, *Flavobacteria* and *Rhizobium*, which are associated with plant growth, were inhibited (Figure 4B). However, *Streptomyces*, *Bacillus*, *Burkholderia*, and *Pseudomonas*, which exert biocontrol effects, were significantly increased in the conditioned rhizosphere soil (Figure 4B). As for bulk soil bacteria, 6 phyla, including 18 genera, showed significant alterations; the relative abundances of 13 genera were significantly decreased, and those of five genera were significantly increased (Supplementary Figure 6).

### Impact of Aboveground Pathogen Infection on Root Exudates of Tea Seedlings

**FIGURE 5:**
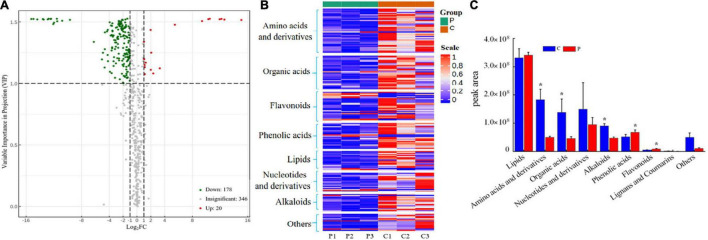
**(A)** Volcano plot of differential expression of metabolites between the two treatments. VIP > 1 indicates that metabolite expression is significantly different. **(B)** Heatmap of significantly differential substances in root exudates of control-treated and pathogen-inoculated tea seedlings. **(C)** Cumulative peak area of compound categories. “C” represents control treatment, “P” represents pathogen infection treatment. Data are means ± SDs of three replicates. *p < 0.05.

Root exudates of healthy and infected tea seedlings were detected and analyzed by UPLC-MS/MS. In total, 544 compounds were identified across all samples (Supplementary Table 7), which could be divided into nine broad categories based on their chemical nature: lipids (102), phenolic acids (73), organic acids (72), amino acids and derivatives (68), flavonoids (66), nucleotides and derivatives (50), alkaloids (41), terpenoids (11), and others (61) (Supplementary Figure 7). All exudates identified were detected in both treatments. However, PCoA showed that the overall exudation pattern of the control tea seedlings differed from that of tea seedlings infected with *Ps. camelliae-sinensis* (Supplementary Figure 8A). A volcano plot showed that the expression of 198 compounds differed significantly (VIP > 1) between the two treatments. The expression of 20 metabolites in the pathogen infection group was significantly upregulated compared to that in the control group, and the expression of 178 metabolites was significantly downregulated (Figure 5A).

When evaluated at the compound level, the 198 significantly altered metabolites included lipids (18), phenolic acids (24), organic acids (32), amino acids and derivatives (42), flavonoids (27), nuclei and derivatives (18), alkaloids (16), and others (21) (Figure 5B and Supplementary Table 7). When evaluated at the group level, amino acids and derivatives, organic acids, and alkaloids were found to be significantly higher (*p* < 0.05) in the control seedlings, whereas phenolic acids and flavonoids were significantly higher in the pathogen-inoculated seedlings (Figure 5C). *Ps. camelliae-sinensis* infection resulted in a significantly higher secretion of phenolic acids and flavonoids, whereas the secretion of amino acids and derivatives, organic acids, and alkaloids was reduced (Figure 5C). The alkaloid putrescine was the exception; it was significantly upregulated in pathogen-inoculated tea seedlings (Supplementary Figure 8B).

### Disease-Suppressive Effect of the Exudate Compounds

**FIGURE 6:**
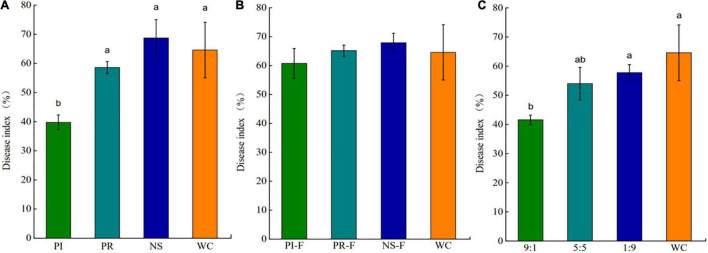
Disease index (DI) of *Ps. camelliae-sinensis-*challenged tea seedlings growing on sterilized soil inoculated with slurries of pathogen-repressed (PR), pathogen-induced (PI), natural soil (NS), and water control (WC) **(A)**, filter-sterilized slurries of PR, PI, NC, and WC **(B)**, and PR and PI mixed at a ratio of 1:9, 5:5, or 9:1, and WC (v/v) **(C)**. Data are means ± SEs of six replicates. a, b, and c indicate significant differences (*p* < 0.01).

Disease-suppressive effect of the exudate compounds showed that the DI of tea seedlings treated with PI slurry was significantly lower than that of tea seedlings treated with PR, NS, or WC slurries (Figure 6A). Importantly, soil slurry filtered to remove the microorganisms had no significant effect on the DI (Figure 6B), indicating that the microorganisms rather than exudate compounds ISR in the tea seedlings. To assess the amount of microbiome required for the induction of ISR, PI slurry was mixed into PR slurry at 90, 50, and 10% w/w. The results showed that 90 and 50% of the PI microbiome could at least partially inhibit the disease (Figure 6C).

### Growth Promotion and Disease Resistance Induction in Tea Seedlings by the Beneficial Microbes

**FIGURE 7:**
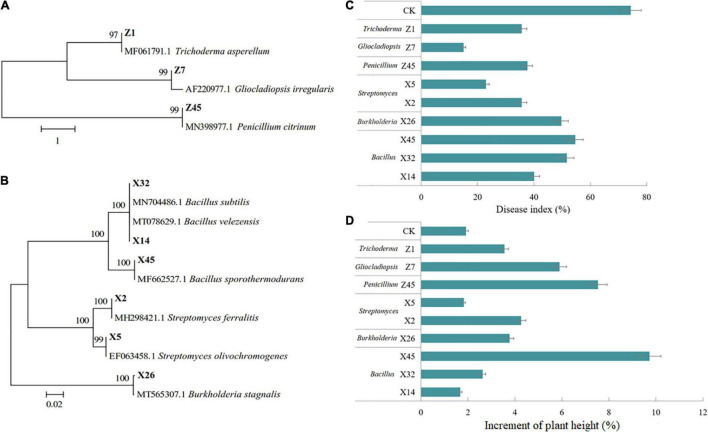
Effect of beneficial fungi and bacteria on tea seedling growth and tea gray blight DI. Hierarchical clustering analysis of *ITS* genes of beneficial fungi **(A)**, and 16S rRNA genes of beneficial bacteria **(B)**. **(C)** DI of tea seedlings grown in soil inoculated with beneficial fungi or bacteria, and challenged by *Ps. camelliae-sinensis* for 7 days. **(D)** Plant height of tea seedlings grown in soil inoculated with beneficial fungi or bacteria for 30 days.

Nine beneficial strains were isolated and screened from the conditioned rhizosphere soil. The three fungi were identified as *Trichoderma asperellum, Gliocladiopsis irregularis*, and *Penicillium citrinum* (Figure 7A), and six bacteria were identified as *Streptomyces ferralitis*, *S. olivochromogenes*, *B. velezensis*, *Burkholderia stagnalis*, *B. subtilis*, and *B. sporothermodurans* (Figure 7B).

The results of confrontation culture showed that all isolates showed antigenic activity toward *Ps. camelliae-sinensis* (Supplementary Table 8). Nine isolates were tested for their ISR-inducing effect on tea seedlings. All nine beneficial microorganisms could reduce the DI of tea seedlings, among which Z7 and X5 had the most obvious effect (Figure 7C). Growth test results showed that all microorganisms except X5 and X14 significantly promoted the growth of tea seedlings, with Z45 and X45 having the most obvious effect (Figure 7D).

## Discussion

Studies have shown that aboveground diseases in plants can affect the performance of their offspring by changing the composition of rhizosphere microorganisms (Yuan et al., 2018); however, different plant diseases caused different alterations in soil microorganisms (Lee et al., 2012; Cha et al., 2016; Gu et al., 2016; Kong et al., 2016; Berendsen et al., 2018). Here, we found that infection of tea seedling leaves by the tea gray blight pathogen can enhance disease defense in their offspring growing in the same soil. Further, we demonstrated that tea gray blight can indirectly promote the growth and disease defense of fresh tea seedlings in the same soil by changing root secretion patterns and recruiting beneficial microorganisms to the rhizosphere.

### Tea Gray Blight Induces Disease Resistance in Tea Plants by Recruiting Beneficial Rhizosphere Microorganisms

Rhizosphere microbial reassembly caused by aboveground pathogen infection has been reported previously. Studies on Arabidopsis (Berendsen et al., 2018; Yuan et al., 2018) and pepper (Lee et al., 2012; Kong et al., 2016) have demonstrated that aboveground pathogen or insect attack can alter the community structure of rhizosphere microorganisms. In this study, aboveground *Ps. camelliae-sinensis* infection caused plant-mediated alterations in fungal and bacterial community structures in the rhizosphere; however, such an effect was not observed in bulk soil. Conversely, Yuan et al. (2018) found that the bacterial structure of bulk soil changed more significantly than that of rhizosphere soil after aboveground pathogen infection. However, this result was likely because these researchers only analyzed microbial communities in bulk soils of the next generation of plants challenged by pathogens, whereas we analyzed tea seedlings challenged by aboveground pathogen infection. Previous research has shown the directional selection of microorganisms by plant roots, and the rhizosphere, which is close to roots, may be more affected by host plants than bulk soil, which supports our results (Bulgarelli et al., 2013; Reinhold-Hurek et al., 2015). Further analysis of the microbial communities at the genus level demonstrated that some genera with potential biocontrol ability, such as *Trichoderma* (Huang et al., 2011), *Penicillium* (Yuan et al., 2021), *Gliocladiopsi*s (Li, 2007), *Pseudomonas* (Trotel-Aziz et al., 2008; Park et al., 2015), *Streptomyces* (Wang, 2014; Chen et al., 2020; Hu et al., 2021), *Bacillus* (Trotel-Aziz et al., 2008; Tahir et al., 2017), and *Burkholderia* (Gao et al., 2015), were increased.

When we isolated these fungi and bacteria and tested their ability to induce ISR and promote growth, we found that nine isolated strains belonging to *Trichoderma*, *Penicillium*, *Gliocladiopsis*, *Pseudomonas*, *Streptomyces*, *Bacillus*, and *Burkholderia* not only induced ISR against *Ps. camelliae-sinensis*, but also promoted the growth of tea seedlings. Overall, these findings indicate that increases in the abundances of these microbes are beneficial to tea seedlings as together, they not only induce ISR, but also promote growth in tea seedlings.

The above findings showed that tea seedlings infected with *Ps. camelliae-sinensis* can recruit specific microbes to aid in their defense. This finding was confirmed in our follow-up research. Tea seedlings were planted in conditioned and control soil and were then challenged with *Ps. camelliae-sinensis*. The results showed that the DI of tea seedlings in conditioned soil was lower than that of tea seedlings grown in the control soil. Recent studies in pepper (Lee et al., 2012; Kong et al., 2016), tomato (Gu et al., 2016), wheat (Dudenhffer et al., 2016), and Arabidopsis (Berendsen et al., 2018; Yuan et al., 2018) support this finding as aboveground pathogen infection triggered changes in rhizosphere microbial communities that ISR in plants. However, the previous studies showed that different foliar pathogens had different effects on rhizosphere microbial communities. A better understanding of the plant genetic basis of disease-induced beneficial root-related microbial recruitment can open up new possibilities for induced resistance research in crops. To our knowledge, this study is the first to demonstrate that tea gray blight can protect tea plants from subsequent aboveground pathogen infection by attracting beneficial microorganisms to the rhizosphere.

### Tea Gray Blight Induces Disease Resistance in Tea Plants by Altering Their Root Exudates, Leading to the Recruitment of Rhizosphere Microorganisms

As for the mechanism of plant-mediated rhizosphere microbial community alterations, previous studies have shown that aboveground pathogen infections regulate rhizosphere microorganisms by altering the plant root exudates (Dudenhffer et al., 2016; Yuan et al., 2018). Further, studies have shown that the interaction mechanisms between different pathogens and plants are completely different (Gu et al., 2016; Hu et al., 2018; Stringlis et al., 2018). In this study, root exudates of tea seedlings infected by *Ps. camelliae-sinensis* were analyzed. The results showed that most of the root exudates, including amino acids and organic acids, were downregulated by aboveground pathogen infection (178 species). This result is inconsistent with findings reported by Yuan et al. (2018) who showed that *P. syringae* pv. *tomato* induced Arabidopsis to secrete amino acids and organic acids. However *Ps. camelliae-sinensis* inhibited the secretion of amino acids and organic acids by tea seedlings. It has been suggested that such different findings may be related to differences in disease progression between pathogens (Camanes et al., 2015) and to metabolic differences between different plant species (Hong et al., 2012; Wang et al., 2016).

More importantly, 20 exudate substances, such as phenolic acids, flavonoids, and putrescine, were significantly upregulated by aboveground pathogen infection. Phenolic acids and flavonoids belong to the phenolic compounds. In fungus–plant interactions, plant phenolic compounds mainly serve as chemical defense substances. Accordingly, when plants are infected by pathogens, plant phenolic compounds accumulate significantly (Wang et al., 2016). Phenolic acids, flavonoids, and putrescine can function as antimicrobial and signaling molecules in the rhizosphere (Verma and Mishra, 2005; Steinkellner et al., 2007; Li et al., 2009). When we evaluated the effects of these compounds in the tea rhizosphere, we found that soil slurry conditioned with the compounds induced by aboveground pathogen infection could reduce the gray blight DI in tea seedlings. This disease resistance-inducing effect was lost in soil slurries from which the microbiome had been removed by filtration, indicating that the microbes, rather than the exudate compounds themselves, elicited resistance in the tea seedlings. Thus, root exudates can induce disease resistance by regulating the community structure of the rhizosphere microorganisms, as also reported by Yuan et al. (2018). This view is supported by research reported by Badri et al. (2013) who showed that sugars and amino acids act as general attractants to a broad range of microbes, whereas phenolic compounds act as specific substrates or signaling molecules for specific microbes, including *Rhizobium*, *Bacillus*, *Sphingomonas*, *Streptomyces*, and *Pseudonocardia*. Therefore, the upregulation of phenolic substances in the tea root exudates may have resulted in the recruitment and aggregation of beneficial microorganisms such as *Pseudomonas*, *Streptomyces*, and *Bacillus* in the tea rhizosphere.

The root exudates of tea plants growing in the natural environment are affected by a myriad of factors. Here, we only analyzed the compounds secreted by roots under controlled conditions and their functions in the rhizosphere. Although we did observe an association between the compounds studied and the functions they performed in the rhizosphere in vivo, further work is needed to clarify the interaction mechanism between secretions and specific microorganisms. Promoting rhizosphere-driven microbial function is a sustainable way to improve the health of tea plants. Exploring the roles of rhizosphere microorganisms in the development of tea plants can help design better disease-resistance strategies. Therefore, further studies are needed to explore the mechanisms of disease resistance induced by specific microorganisms and to reveal the pathways of signal transduction between species in the rhizosphere.

## Conclusion

Aboveground infection by *Ps. camelliae-sinensis* triggers disease resistance to subsequent infection in tea seedlings by altering root exudates, leading to the recruitment of beneficial microbes in the rhizosphere. This ISR was mediated by different fungal and bacterial communities in soil pretreated with exudates from plants with aboveground pathogen infection. When tea seedlings were challenged with *Ps. camelliae-sinensis*, their root secretion profile changed, and the secretion of phenolic acids and flavonoids increased. The addition of a mixture of phenolic acids and flavonoids to the soil led to the induction of a response similar to that caused by aboveground pathogen infection. In other words, *Ps. camelliae-sinensis* infection can alter the root secretion of tea seedlings to recruit beneficial microorganisms to the rhizosphere and induce plants to produce systemic disease resistance.

## Data Availability Statement

The datasets presented in this study can be found in online repositories. The names of the repository/repositories and accession number(s) can be found below: NCBI (accession: PRJNA774068).

## Author Contributions

XH and LY conceived the study and directed the project. QW and RY performed the microbial isolation. QW and YY performed the microbial identification. WP, XM, and QW performed the root exudates analysis. AJ, WZ, and LL performed the microbial diversity sequencing and analysis. QW and PL performed all the tea seedling culture and inoculation experiments. XH, LY, and QW wrote the manuscript. All authors contributed to the article and approved the submitted version.

## Conflict of Interest

The authors declare that the research was conducted in the absence of any commercial or financial relationships that could be construed as a potential conflict of interest.

## Publisher’s Note

All claims expressed in this article are solely those of the authors and do not necessarily represent those of their affiliated organizations, or those of the publisher, the editors and the reviewers. Any product that may be evaluated in this article, or claim that may be made by its manufacturer, is not guaranteed or endorsed by the publisher.
