# Supplementary material for: Tea Plants With Gray Blight Have Altered Root Exudates That Recruit a Beneficial Rhizosphere Microbiome to Prime Immunity Against Aboveground Pathogen Infection
Source: Front Microbiol. 2021 Dec 1;12:774438. doi: 10.3389/fmicb.2021.774438 (PMC8672095; doi:10.3389/fmicb.2021.774438)
Supplement: Supplementary file 1 [file Table_3.DOC]

SUPPLEMENTARY MATERIAL


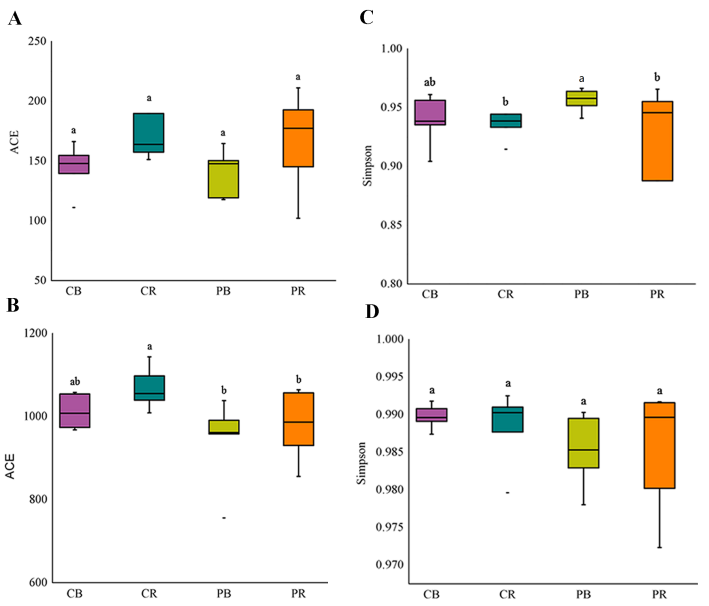


Figure S1 Abundance and diversity of soil fungi (A,C) and bacteria (B,D)


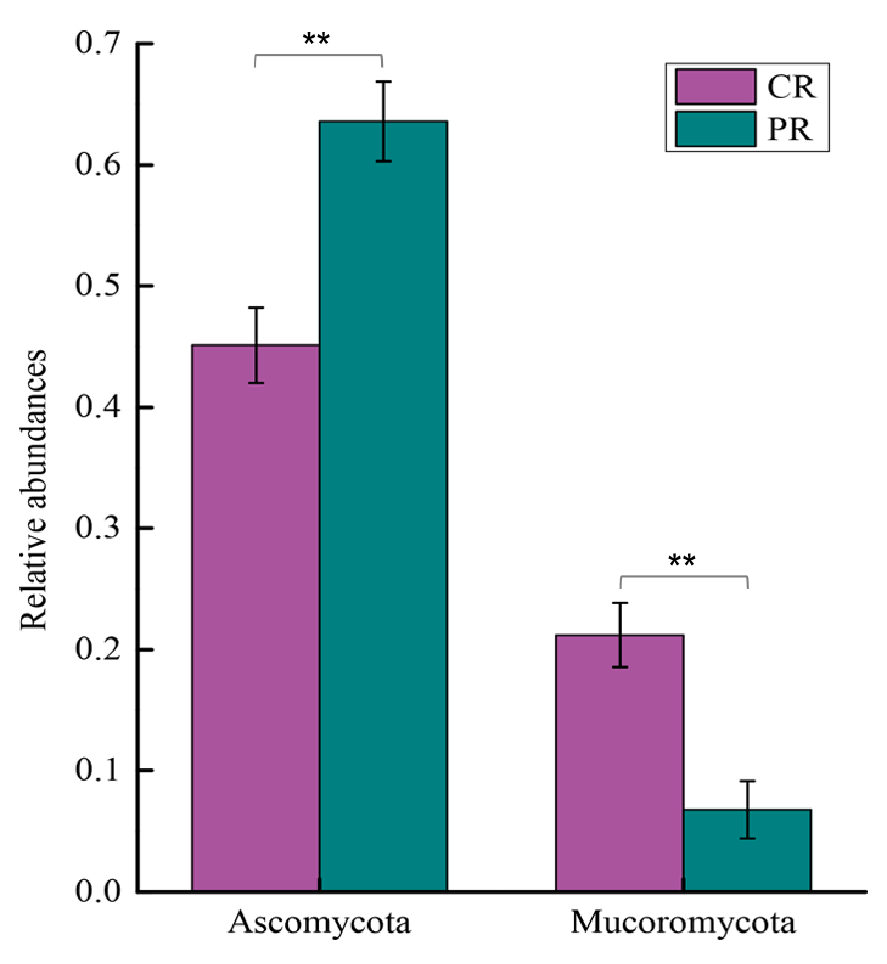


Figure S2. Difference of fungi between conditioned and control rhizosphere soil at the phylum level. Data presented represent mean ± SE (n=6). ***p* < 0.01 versus control soil.


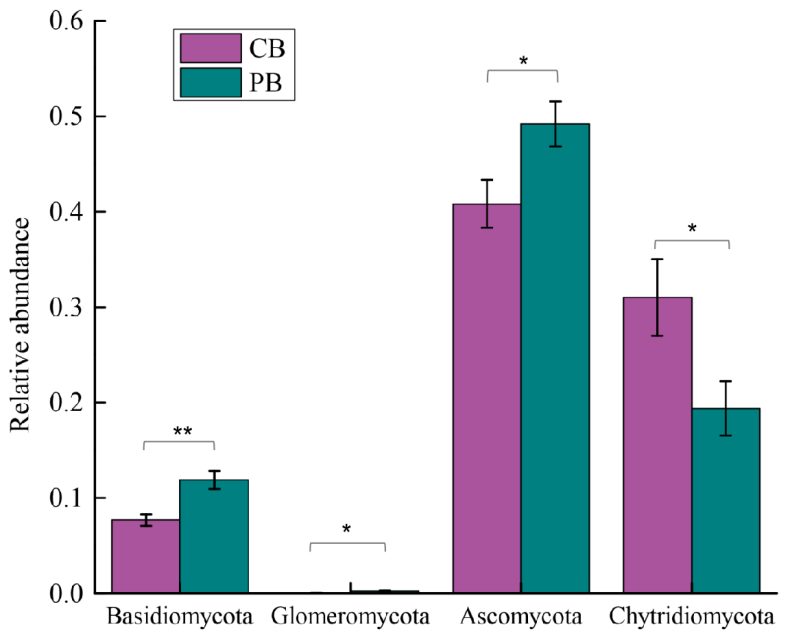


Figure S3. Difference of fungi between conditioned and control bulk soil at the phylum level. Data presented represent mean ± SE (n=6). *p < 0.05, **p < 0.01 versus control soil.


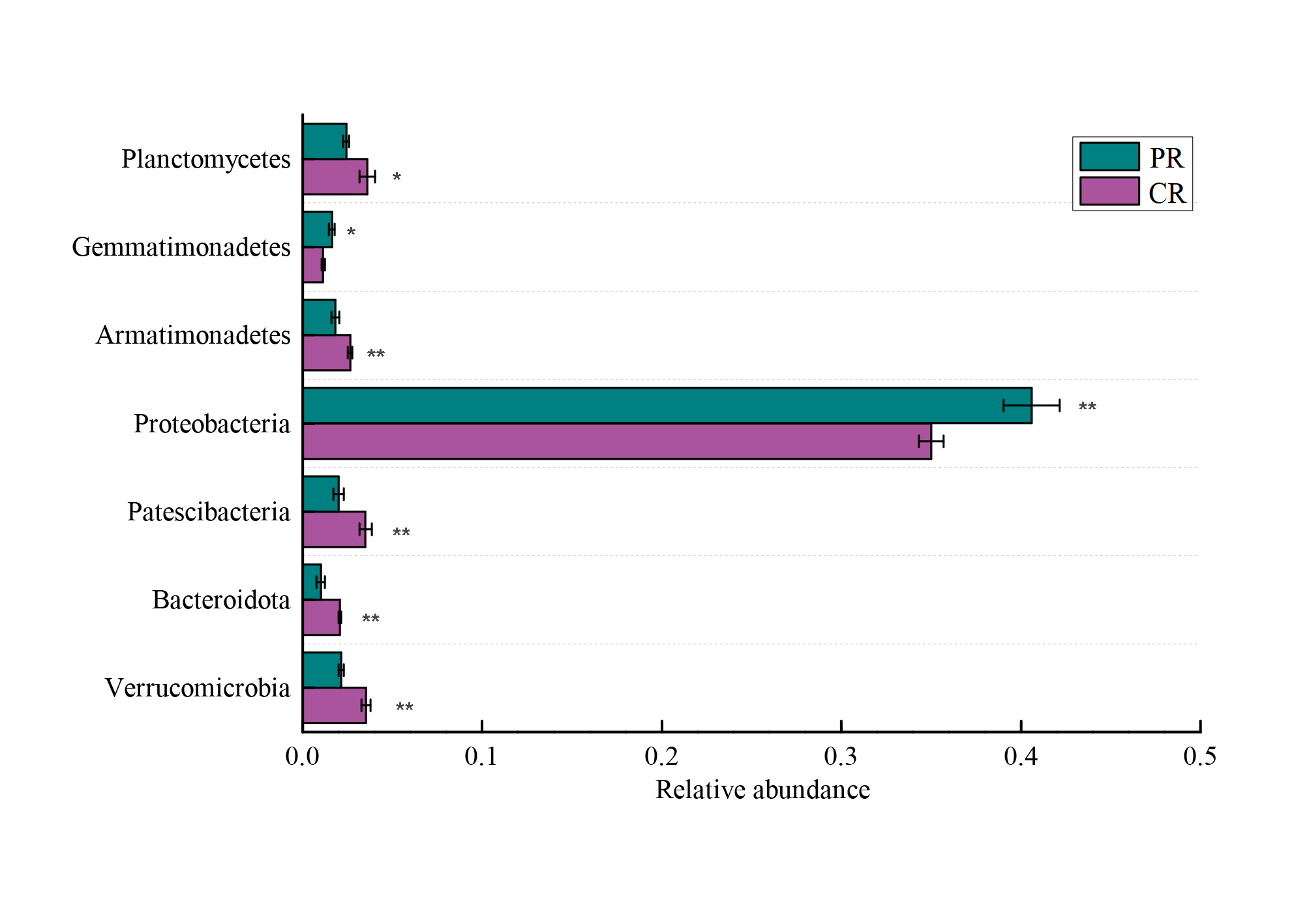


Figure S4. Difference of bacteria between conditioned and control rhizosphere soil at the phylum level. Data presented represent mean ± SE (n=6). *p < 0.05, **p < 0.01 versus control soil.


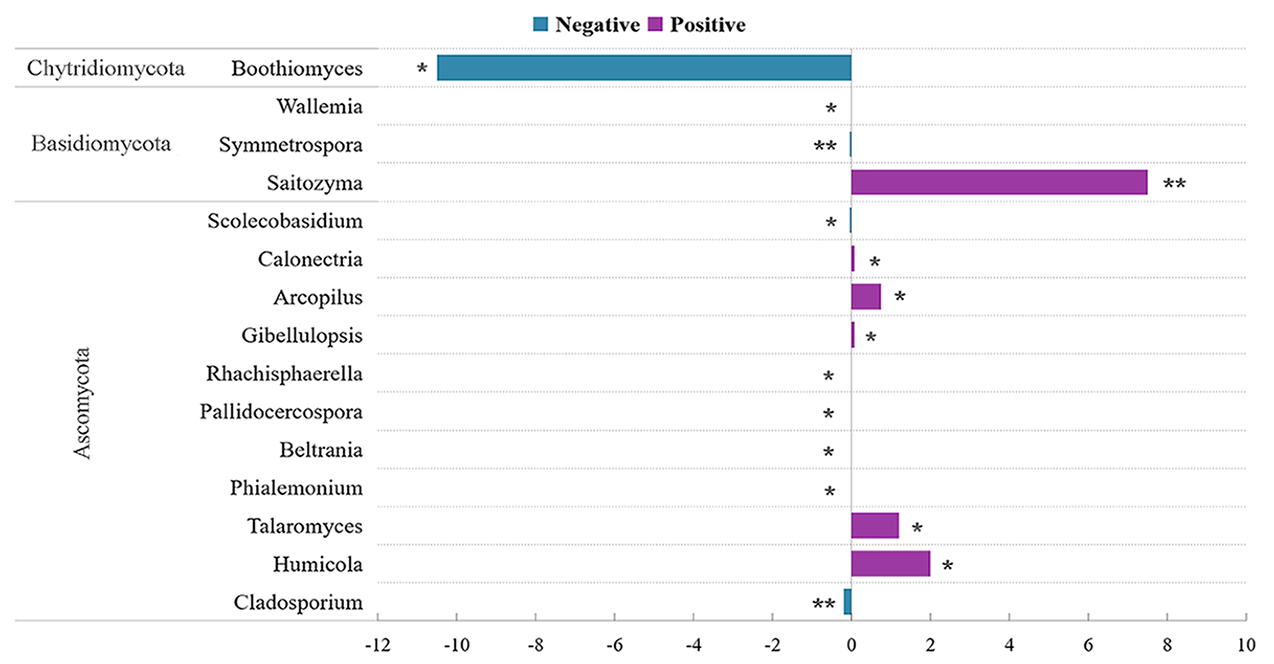


Figure S5. Difference of fungi between conditioned and control bulk soil at the genus level. Data are means ± SEs (n=6). **p* < 0.05, ***p* < 0.01 versus control soil.


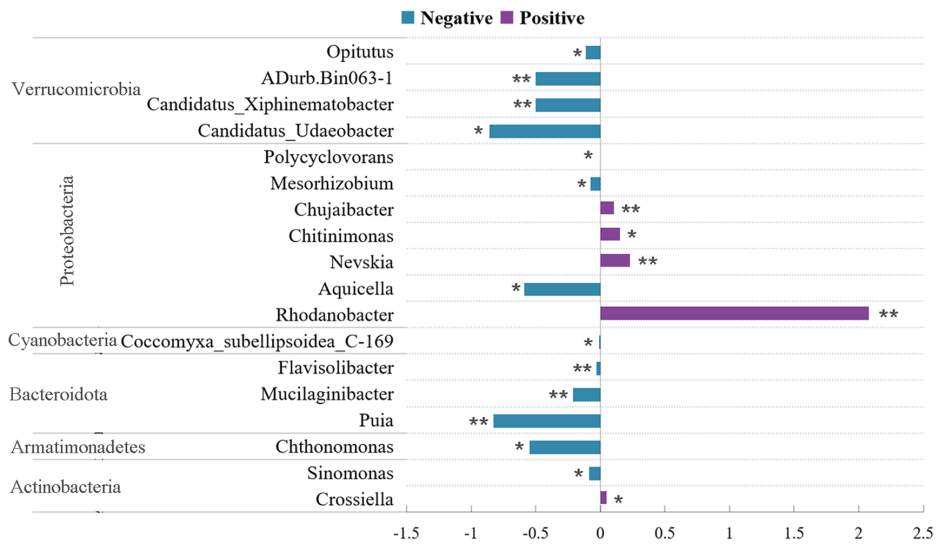


Figure S6. Difference of bacteria between conditioned and control bulk soil at the genus level. Data presented represent mean ± SE (n=6). **p* < 0.05, ***p* < 0.01 versus control soil.


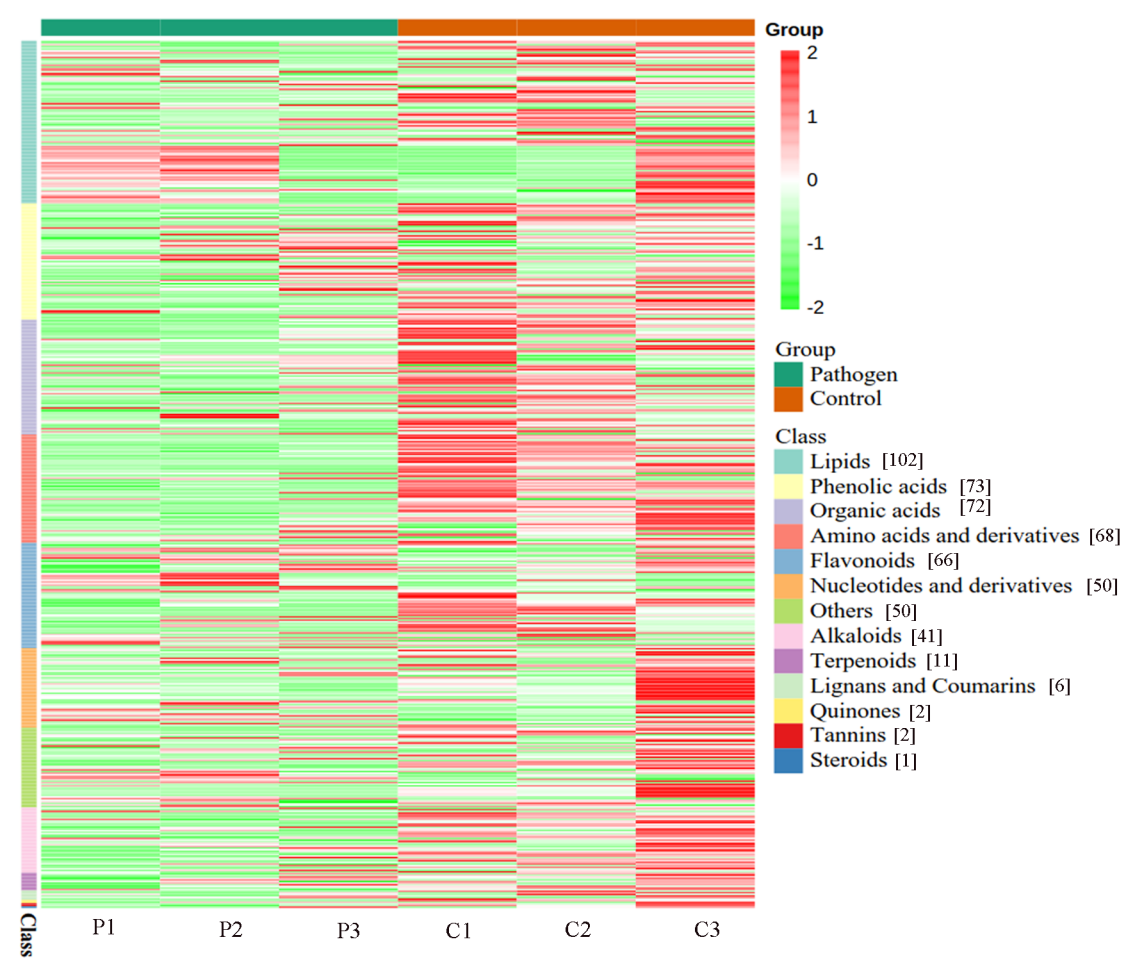


Figure S7. Heat map of root exudates between pathogen infection and control tea seedlings. “C” represents control treatment, “P” represents pathogen infection treatment.


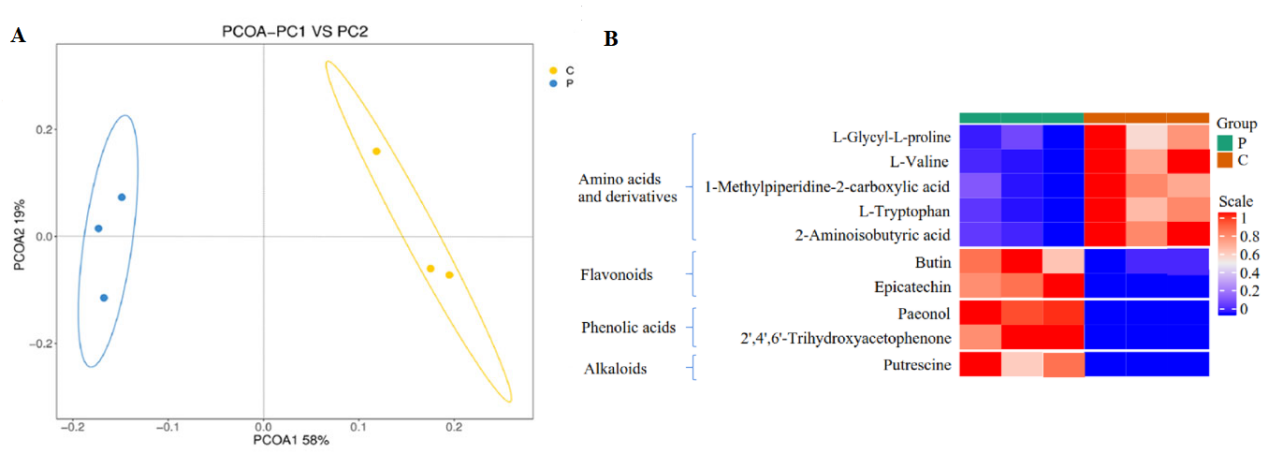


Figure S8 PoCOA analysis of root exudates (A) and heat map of representative compounds (B)

Table S1 Primer pairs used to amplify gene regions of Pestalotiopsis-like species.

| Gene | Gene sequence | Reference |
| --- | --- | --- |
| ITS | CTTG GTCA TTTA GAGG AAGT AA | Ruijuan Yang et al，2011 |
|  | TCCT CCGC TTAT TGAT ATGC |  |
| β-tubulin | 5′-GGTAACCAAATCGGTGCTGCTTTC-3′ | Zhenhua Wang et al，2017 |
|  | 5′-ACCCTCAGTGTAGTGACCCTTGGC-3′ |  |
| TEF | AGTGCGGTGGTATCGACAAG | Dongxue LI et al，2018 |
|  | TTGCCCTCCTTCTTGACACC |  |

Tables S2 Disease grade standard of tea gray blight leaves.

| Disease grade | Degree of disease | Representative value |
| --- | --- | --- |
| Class one | No disease spots | 0 |
| Class two | The leaf surface was dark and spots were rare. The diameter of the spots were ˂ 0.4 cm | 1 |
| Class three | The surface of the leaves had many disease spots, but few were contiguous. The diameter of the spots were ≥ 0.4 cm and ˂ 0.7 cm | 2 |
| Class four | There are many and continuous disease spots on the surface of leaves. The diameter of the spots were ≥ 0.7 cm and ˂ 1 cm | 3 |
| Class five | The diseased spots on the surface of leaves were contiguous, and The diameter of the spots were ≥ 1 cm | 4 |

Table S3 PCR amplification primers of beneficial microorganisms

| Gene | Primer name | Sequence (5′→3′) |
| --- | --- | --- |
| ITS (ITS1) | ITS1 | 5′-CTTGGTCATTTAGAGGAAGTAA-3′ |
|  | ITS4 | 5′-TCCTCCGCTTATTGATATGC-3′ |
| 16S（V3-V4） | F27 | 5′-AGAGTTTGATCMTGGCTCAG-3′ |
|  | R1492 | 5′-TACGGYTACCTTGTTACGACTT-3′ |

Table S4 Chemical properties of pathogen conditioned soil and control soil

|  | pathogen-conditioned soil | control soil | P value |
| --- | --- | --- | --- |
| PH | 4.79±0.14 | 4.87±0.11 | 0.90 |
| AK(mg/kg) | 52.37±0.65 | 53.37±1.12 | 0.12 |
| AP(mg/kg) | 8.66±0.10 | 8.44±0.10 | 0.17 |
| NO3−(mg/kg) | 37.50±1.18 | 36.63±1.14 | 0.16 |
| NH4+(mg/kg) | 35.50±0.82 | 36.40±0.75 | 0.72 |

Table S5 Processed sample data information to analyze fungal community

| Sample ID | Raw CCS | Clean CCS | Effective CCS | AvgLen(bp) | Number of sequences | Number of OTUs |
| --- | --- | --- | --- | --- | --- | --- |
| CB1 | 7413 | 7312 | 7289 | 566 | 7201 | 111 |
| CB2 | 7376 | 7253 | 7238 | 587 | 7109 | 148 |
| CB3 | 7489 | 7353 | 7350 | 585 | 7136 | 138 |
| CB4 | 7384 | 7266 | 7244 | 582 | 7040 | 165 |
| CB5 | 7494 | 7375 | 7360 | 577 | 7226 | 148 |
| CB6 | 7502 | 7411 | 7395 | 567 | 7288 | 141 |
| CR1 | 7504 | 7374 | 7334 | 604 | 7084 | 181 |
| CR2 | 7618 | 7249 | 6832 | 584 | 4862 | 223 |
| CR3 | 7538 | 7403 | 7392 | 600 | 7243 | 158 |
| CR4 | 7544 | 7402 | 7392 | 596 | 7235 | 144 |
| CR5 | 7440 | 7300 | 7297 | 601 | 7158 | 156 |
| CR6 | 7595 | 7438 | 7421 | 603 | 7250 | 153 |
| PB1 | 7545 | 7448 | 7442 | 589 | 7333 | 117 |
| PB2 | 7608 | 7502 | 7490 | 579 | 7379 | 119 |
| PB3 | 7475 | 7347 | 7331 | 587 | 7144 | 149 |
| PB4 | 7245 | 7114 | 7107 | 580 | 6988 | 147 |
| PB5 | 7398 | 7261 | 7254 | 584 | 7094 | 145 |
| PB6 | 7601 | 7465 | 7437 | 579 | 7264 | 161 |
| PR1 | 7603 | 7462 | 7380 | 604 | 7076 | 180 |
| PR2 | 7525 | 7379 | 7368 | 594 | 7190 | 138 |
| PR3 | 7400 | 7263 | 7235 | 584 | 6975 | 203 |
| PR4 | 7508 | 7361 | 7219 | 592 | 7060 | 170 |
| PR5 | 7547 | 7415 | 7392 | 590 | 7224 | 155 |
| PR6 | 7435 | 7310 | 7297 | 602 | 7213 | 101 |
| Sum | 179787 | 176463 | 175496 |  | 169772 | 3651 |

Table S6 Processed sample data information to analyze bacterial community

| Sample ID | Raw CCS | Clean CCS | Effective CCS | AvgLen  (bp) | Number of sequences | Number of OTUs |
| --- | --- | --- | --- | --- | --- | --- |
| CB1 | 7376 | 6384 | 6368 | 1442 | 5634 | 784 |
| CB2 | 7368 | 6398 | 6387 | 1440 | 5677 | 783 |
| CB3 | 7398 | 6322 | 6309 | 1441 | 5507 | 810 |
| CB4 | 7445 | 6382 | 6361 | 1442 | 5438 | 831 |
| CB5 | 7460 | 6471 | 6459 | 1441 | 5807 | 803 |
| CB6 | 7545 | 6478 | 6465 | 1441 | 5703 | 803 |
| CR1 | 7514 | 6389 | 6332 | 1442 | 5416 | 760 |
| CR2 | 7354 | 6302 | 6247 | 1440 | 5533 | 775 |
| CR3 | 7488 | 6438 | 6412 | 1442 | 5340 | 855 |
| CR4 | 7519 | 6511 | 6484 | 1443 | 5917 | 744 |
| CR5 | 7574 | 6460 | 6415 | 1442 | 5497 | 783 |
| CR6 | 7477 | 6387 | 6350 | 1441 | 5624 | 733 |
| PB1 | 5295 | 4593 | 4588 | 1447 | 4028 | 593 |
| PB2 | 7492 | 6512 | 6500 | 1439 | 5889 | 740 |
| PB3 | 7433 | 6401 | 6374 | 1441 | 5656 | 772 |
| PB4 | 7561 | 6541 | 6530 | 1441 | 5813 | 783 |
| PB5 | 7465 | 6448 | 6434 | 1439 | 5825 | 750 |
| PB6 | 7462 | 6451 | 6438 | 1440 | 5735 | 794 |
| PR1 | 7557 | 6502 | 6462 | 1443 | 5687 | 723 |
| PR2 | 7459 | 6456 | 6438 | 1442 | 5871 | 730 |
| PR3 | 7499 | 6427 | 6403 | 1441 | 5470 | 786 |
| PR4 | 7498 | 6500 | 6482 | 1443 | 6016 | 658 |
| PR5 | 7532 | 6502 | 6465 | 1441 | 5775 | 789 |
| PR6 | 7506 | 6443 | 6426 | 1440 | 6016 | 597 |
| Sum | 177277 | 152698 | 152129 |  | 134874 | 18179 |

Table S7 Species of compounds secreted by the roots of control plants and infected by pathogens

| Class I | Number of compounds | Up | Down |
| --- | --- | --- | --- |
| Lipids | 102 | 2 | 16 |
| Phenolic acids | 73 | 2 | 22 |
| Organic acids | 72 | 1 | 31 |
| Amino acids and derivatives | 68 | 1 | 41 |
| Flavonoids | 66 | 9 | 18 |
| Nucleotides and derivatives | 50 | 2 | 16 |
| Alkaloids | 41 | 1 | 15 |
| Terpenoids | 11 | 0 | 0 |
| Others | 61 | 2 | 19 |
| **Total** | **544** | **20** | **178** |

Table S8 Antagonistic effect of microbial isolates against the pathogen of tea gray blight

| Kingdom | Genus | Number | Inhibition rate(%) |
| --- | --- | --- | --- |
| Fungus | *Trichoderma* | Z1 | 38.51±2.43 |
|  | *Gliocladiopsis* | Z7 | 20.35±2.43 |
|  | *Penicillium* | Z45 | 21.65±1.92 |
| Bacterium | *Streptomyces* | X2 | 43.32±2.10 |
|  | *Streptomyces* | X5 | 34.84±3.23 |
|  | *Bacillus* | X14 | 20.69±3.42 |
|  | *Burkholderia* | X26 | 16.58±5.09 |
|  | *Bacillus* | X32 | 25.90±5.05 |
|  | *Bacillus* | X45 | 40.89±1.58 |
